# Possibilities of Managing Waste Iron Sorbent FFH after CO_2_ Capture as an Element of a Circular Economy

**DOI:** 10.3390/ma17112725

**Published:** 2024-06-04

**Authors:** Tomasz Kamizela, Mariusz Kowalczyk, Małgorzata Worwąg, Katarzyna Wystalska, Magdalena Zabochnicka, Urszula Kępa

**Affiliations:** Faculty of Infrastructure and Environment, Czestochowa University of Technology, J.H. Dąbrowskiego 69, 42-201 Częstochowa, Poland; tomasz.kamizela@pcz.pl (T.K.); mariusz.kowalczyk@pcz.pl (M.K.); katarzyna.wystalska@pcz.pl (K.W.); urszula.kepa@pcz.pl (U.K.)

**Keywords:** FFHCO2, waste, coagulation, dewatering, bioleaching

## Abstract

With a growing need to reduce greenhouse gas emissions, innovative carbon dioxide sorbents are being sought. One of the sorbents being tested is nanoparticle ferric hydrosol (FFH). In parallel with sorbent testing, it is also necessary to test the used sorbent after carbon dioxide capture (FFHCO2) and to develop an optimal method for its processing and management. The research described in this article evaluated the potential use of FFHCO2 in dewatering, coagulation and bioleaching processes. The research results indicate that the basic strategy for dealing with waste FFHCO2 sorbent should be to minimize the amount of waste by volume reduction—dewatering. Recycling of FFHCO2 as an iron waste coagulant or its processing products by bioleaching had no technological justification. It is only proposed to recover the material—iron compounds—if it is environmentally and economically justified.

## 1. Introduction

With an increasingly urgent need to reduce greenhouse gas emissions, the search for effective methods of capturing carbon dioxide is becoming a priority for the global scientific and industrial community. In recent years, the development of innovative sorbents capable of effectively absorbing CO_2_ from various sources has become an area of intense research. From traditional sorption materials to advanced nanomaterials, the variety of solutions available offers hope for the creation of effective, economical and sustainable CO_2_ capture technologies [1,2,3].

One of the tested sorbents is iron sorbent—nanoparticle ferriferous hydrosol (FFH) (EUREKA/InnoCO2Sorbent/2/2021—Capture of carbon dioxide by innovative sorbent InnoCO2Sorbent). FFH contains Fe(II) and Fe(III) and its action is mainly through sorption, coagulation and reduction mechanisms [4]. There are several reasons why iron compounds can be attractive sorbents. The main reason is the availability of raw materials; iron is one of the most abundant elements on Earth. The second is chemical reactivity: Iron compounds can react with CO_2_ to form stable chemicals, making them potentially effective sorbents. In addition, some iron compounds can form stable complexes with CO_2_, which means they can effectively capture and store CO_2_ for longer periods of time. The ability to regenerate is also important. Some iron-based sorbents can be regenerated, meaning they can be reused after a long period of use, which is important for the economics of the process [5,6]. The carbon dioxide capture process, regardless of the technology used, produces spent sorbents that become potential waste products. The effectiveness of actions to reduce CO_2_ emissions must be balanced with waste management to ensure a sustainable approach to environmental protection [7,8,9].

A fundamental priority in waste management, including the management of used sorbents, is to avoid and/or reduce the amount of waste. It is necessary to implement carbon dioxide capture processes using more effective technologies than commonly used [10,11,12]. For waste already generated, the priority is to maximize the recovery of valuable raw materials from used sorbents through, e.g., recycling, reusing or energy recovery. Recycling processes may include the recovery of iron or other valuable components from sorbents and their reuse in industrial or municipal processes [13,14]. Additionally, it is important that waste (used) sorbents are properly managed to minimize their impact on the environment and public health and to be economically viable [15,16]. This may include controlling emissions of harmful substances, preventing soil and water pollution as well as ensuring the safe disposal of waste [17,18,19]. Such an approach is directly linked to circular economy principles that are focused on developing processes that can bring benefits to the environment, society and economy [20,21].

Nowadays, carbon dioxide is captured mainly by chemical absorption methods (e.g., primary amines), physical absorption methods (e.g., pressure swing absorption and vacuum pressure swing absorption) or by solid sorbents (a solid CaO sorbent derived from natural limestone). Such absorption methods are costly and highly energy consuming [6,22]. Metal oxides, such as, e.g., CaO, MgO and FeO can react with CO_2_ and are nowadays very promising sorbents because they can be carbonated and regenerated several times [5]. Ferriferous hydrosol (FFH) nanoparticles are generated by the electrolysis of wasted iron scrap by-products. Low-cost FFH sorption material is mainly made of Fe(II) and Fe(III) and iron particles [4]. Mendoza et al. [5] also investigated carbon dioxide capture/release reactions using iron-based sorbents. However, they used magnetite Fe_3_O_4_ and hematite Fe_2_O_3_ as mixtures of raw materials used in metallurgy. The main difference from the current work on FFH is that sorbent materials were used in four carbonation and calcination cycles. This approach showed good stability of CO_2_ absorption capacity. As a result, they concluded that siderite or iron oxides are potential and effective reversible sorbents for CO_2_ capture.

Used CO_2_ capture sorbents can be regenerated, reused, recycled or stored. Regeneration involves reversing the absorption process so that the sorbent can be reused. Disposal includes incineration or landfill when regeneration is uneconomical. Recycling involves the processing of used materials to recover valuable components or to produce new sorbents [23,24,25]. Recycling of sorbents using biological methods can be recommended in the context of a circular economy, as it allows for the sustainable management of used materials. Biological methods, such as bioleaching or the use of sewage sludge technology, can effectively process sorbents, reducing their toxicity and enabling the recovery of valuable components. This type of approach minimizes waste, reduces the need for primary raw materials and supports sustainable development by closing the life cycle of materials [15,16,17,18,19].

The effectiveness of iron-based sorbents for CO_2_ capture depends on many factors, such as the type of iron compound, operating conditions (e.g., temperature, pressure) and structural properties of the sorbent. In parallel with testing the new FFH sorbent, testing of the used sorbent after capturing carbon dioxide FFHCO2 is also required. In the field of research on used sorbents, it is important to use physical, chemical and biological processes to develop the most advantageous disposal method. This article has assessed the potential usefulness of using FFHCO2 in dewatering, coagulation and bioleaching processes. Due to the characteristics of the used sorbent, these processes were considered the most appropriate for the recovery, recycling and utilization of FFHCO2.

## 2. Materials and Methods

### 2.1. Research Stages

**Figure 1:**
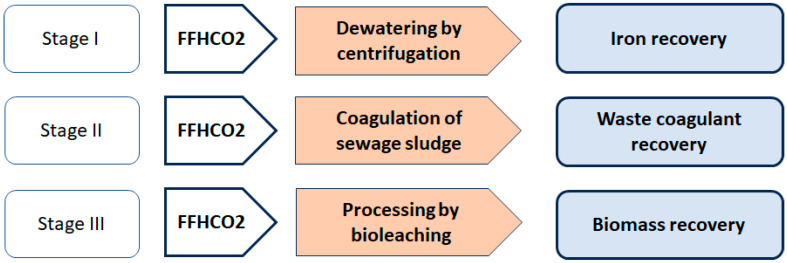
Research stages of processing of waste FFHCO2 sorbent.

Three stages of research were carried out to investigate the possibility of managing the waste sorbent FFHCO2 (Figure 1).

In stage I, the possibility of effectively separating the solid phase (iron compounds) using the centrifugation process was determined. The basic task in waste processing is to remove water from the waste, which enables raw materials to be recovered.

Stage II consisted in determining the suitability of FFHCO2 as an iron coagulant. FFHCO2 was dosed as a conditioning agent for sewage sludge before dewatering. To compare the dewatering effects, the commercial iron coagulant PIX (Kemira, Poland) 113 was used.

Stage III concerned the processing of FFHCO2 by bioleaching and determining the suitability of the substrate as a growth medium for the bioleaching bacteria *Acidithiobacillus thiooxidans* and *Acidithiobacillus ferrooxidans*. It was assumed that the produced acidic biomass could be used as a fertilizer substrate.

### 2.2. The FFHCO2 Substrate

**Figure 2:**
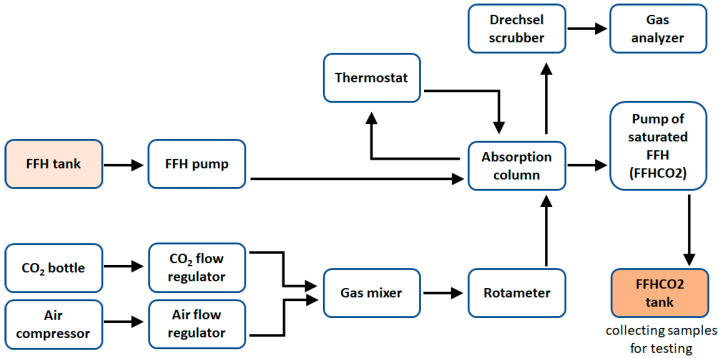
Diagram of a laboratory installation for CO_2_ capture.

Nanoparticle ferriferous hydrosol (FFH) is an aqueous solution of iron II hydroxide. The content of total iron in FFH is 30 g/L. The content of iron Fe (II) in this sorbent is 24 g/L and the content of iron chloride is 4 g/L. The pH of FFH is pH 8.0. The research used waste iron sorbent (FFHCO2) generated after carbon dioxide capture in a laboratory installation. The installation consisted of a gas preparation module, an absorption column, a gas analyzer and a thermostat maintaining the set process temperature. Gas with a given CO_2_ concentration was prepared by mixing pure carbon dioxide (from a gas cylinder) with air (air compressor). A proper gas ratio and flow were achieved by using electronic flow regulators. Gas streams were mixed in a gas mixer. The mixer was a 500 mL glass container with an inert glass filling (Raschig rings). The role of the absorber was played by glass columns with an internal diameter of 42 mm and a total length of 1000 mm, equipped with a sieve bottom (located on the narrowing) on which a glass filling in the form of Raschig rings was placed. Saturated FFH (FFHCO2) was collected using a pump in a tank from which samples were taken for testing (Figure 2).

**Figure 3:**
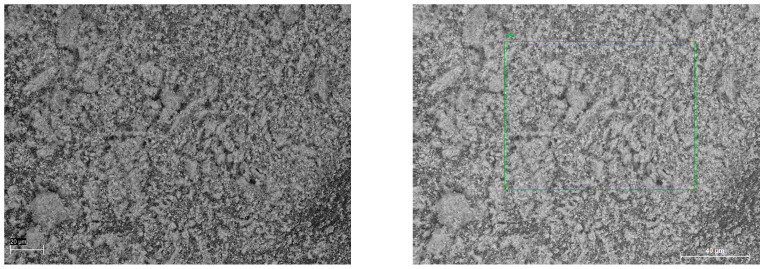
FFH images from a scanning electron microscope (20 µm—left, 40 µm—right).

FFH has a very large surface with chemically active sites, which can react with gaseous molecules of carbon dioxide (Figure 3).

We propose the following carbonation reaction of FFH:

Fe(OH)_2_ + CO_2_ → FeCO_3_ + H_2_O

The above chemical reaction allows the production of iron(II) carbonate: siderite.

The test substrate FFHCO2 showed magnetic properties, which was due to significant iron content (Table 1).

### 2.3. Materials and Reagents Used in Stage I Research

In Stage I, only the FFHCO2 substrate was tested. No dosing of reagents was used. A centrifugation process was used to reduce the hydration of FFHCO2. The centrifugation times were 1 and 2 min. The relative centrifugal force (rcf) was 1000, 3000, 5000, 7000 and 9000.

### 2.4. Materials and Reagents Used in Stage II Research

PIX 113 is an aqueous solution of Fe_2_(SO_4_)_3_. The coagulant has the following parameters: iron III sulphate concentration 35–50%, iron II sulphate concentration 0.1–1.5%, magnesium sulphate concentration <0.25%, dynamic viscosity about 30 mPas and density 1.50–1.58 g/mL. Sewage sludge from a municipal treatment plant with a capacity of 200 m^3^∙d^−1^ was used for Stage II research. The sludge was produced as a result of mechanical and biological treatment and aerobic stabilization for 25 days. The basic physical and chemical parameters of the collected sludge are presented in Table 2. 

### 2.5. Materials and Reagents Used in Stage III Research

#### 2.5.1. Cultivation of Bioleaching Bacteria in Stage III

*Acidithiobacillus thiooxidans* and *Acidithiobacillus ferrooxidans* are the most commonly used bacteria in bioleaching. To produce energy, *A. thiooxidans* oxidizes sulfur compounds to sulfuric acid. *A. ferrooxidans* oxidizes Fe^+2^ to Fe^+3^ using iron and sulfur compounds to produce energy. At an even lower pH of pH 1.0–3.0, the formation of sulfuric acid allows the solubilization of metals. It has been found that low pH does not negatively affect the growth and metabolism of *A. thiooxidans* and *A. ferrooxidans* [26,27,28]. The cultivation of bioleaching bacteria was carried out at a temperature of 20 °C. The optimal temperature for the growth of acidic bacteria *A. thiooxidans* and *A. ferrooxidans* is 30 °C [26,27,28]. Lowering the culture temperature resulted from the assumption of reducing energy demand (thermal energy) during the cultivation of bioleaching bacteria.

The cultivation of bioleaching bacteria was carried out on municipal sewage sludge according to methodology proposed by Li et al. [22]. A 9 K medium was used for bacterial cultivation with the following composition: 3 g/L (NH_4_)_2_SO_4_, 0.5 g/L MgSO_4_·7H_2_O, 0.5 g/L K_2_HPO_4_·3H_2_O, 0.1 g/L KCl, 0.01 g/L Ca(NO_3_)_2_, 10 g/L elemental sulfur (S^0^), 44 g/L FeSO_4_·7H_2_O and 1 L distilled water.

#### 2.5.2. Inoculation (Stage III)

After five cycles of 7-day incubations, the cultured bacterial suspension was separated from the solution by centrifugation. Centrifugation was performed at 3000 rcf for 2 min. Biomass separated in the centrifugation process was dissolved in 1.0 L of K9 medium. A bacterial suspension of *A. thiooxidans* and *A. ferrooxidans* in K9 medium solution was used to inoculate the samples. This was due to the fact that the tested substrate FFHCO2 did not contain the nutrients necessary for the growth of bioleaching bacteria (Table 3).

#### 2.5.3. Research Combinations (Stage III)

**Figure 4:**
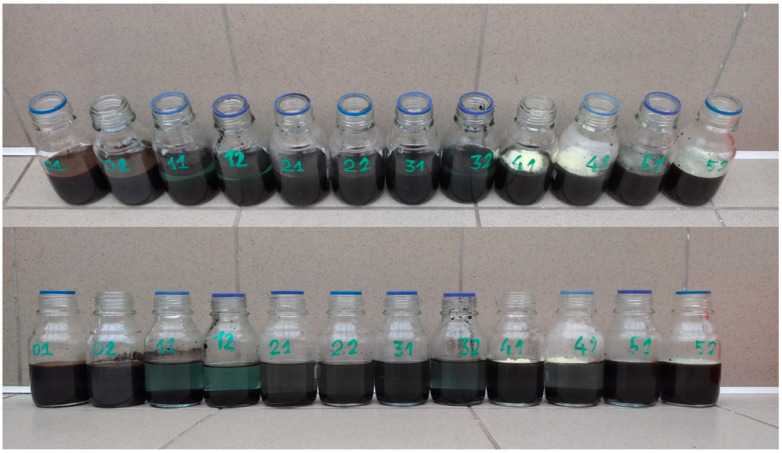
Samples for bioleaching before 21-day incubation in stage III.

Experiments were performed as 21-day batch cultures. Reactors with an active volume of 0.15 L were mixed and aerated by rotation (180 rpm). The process temperature was set at 20 °C. The tests were performed in six combinations (A–F), (Table 4). The combinations included the addition of inoculum and conditions determining the growth of bioleaching bacteria: pH correction to 2.0, addition of 10.0 g/L of elemental sulfur and addition of 44.0 g/L of iron II sulfate. Combinations A and B were control combinations (without and with sample acidification). Combinations C–F used the inoculation consisting of bacterial biomass suspended in pure K9 medium. FeSO_4_ · 7H_2_O and S^0^, in doses optimal for growth, were energy substrates for microorganisms. The samples were thermostated at 20 °C. The tests were performed in two repetitions (Figure 4).

### 2.6. Methodology

The parameters of the samples were tested according to the following:

1. dry solids (DS) [29].
2. volatile solids (VS), [30].
3. capillary suction time (CST), [31].
4. specific resistance of filtration (SRT), [32].
5. pH was determined using the potentiometric method (Elmetron CPC-505, Zabrze, Poland).
6. Conductance (C) was determined using direct conductivity (Hydromet CD-210 probe, Gliwice, Poland).
7. Reduction–oxidation potential (redox) was determined by an electrochemical method (Hydromet ERPt—11X probe, Gliwice, Poland).
8. Alkalinity was determined by titration using a pH meter in accordance with the standard [33].
9. The content of sulfur and iron compounds was determined using cuvette tests for a HACH DR 6000 spectrophotometer (Hach Company, Loveland, CO, USA); LCK 153—sulfates, LCK, LCK 321—iron, and LCK 654—sulfites.
10. The turbidity (T) of the filtrate after separation of the solid phase in the vacuum filtration process was determined using a Hach 2100N turbidity meter (Hach Company, Loveland, CO, USA).
11. The efficiency of phase separation by a centrifugation (Centrifugation efficiency, CE) method was determined using the following formula:

(1) $$CE=\frac{DS-{DS}_{SUPERNATANT}}{DS}\times 100\%$$

where DS—dry solids of the centrifuged sample and ${DS}_{SUPERNATANT}$—dry solids of the supernatant after centrifugation

### 2.7. Statistical Analysis

Statistical analysis was carried out by STATISTICA software (STATISTICA 13.3, TIBCO Software Inc., Palo Alto, CA, USA). Analysis of variance (ANOVA) was used to test the significance of differences. A Tukey test was used for statistically significant data *p* < 0.05 (post-hoc analysis). Results of the analysis made it possible to distinguish groups (marked with letters a, b) between which there was a statistically significant difference. Test results assigned to the same group (e.g., a) did not show statistically significant differences.

## 3. Results and Discussion

### 3.1. Results of Stage I Tests

The waste iron sorbent was a very highly hydrated suspension. The use of centrifugation as a method of sludge–supernatant phase separation was an efficient solution. Centrifugation for 1 min at the lowest overload of rcf 1000 was already very effective. The sludge after centrifugation contained almost 99% of the previously suspended solids. Only 1% of the solids remained in the supernatant after centrifugation (Table 5). Analysis of the CE value suggests that the centrifugation time did not affect the separation efficiency. This was confirmed by a static analysis, which showed that there were no significant differences between the centrifugation times of 1 and 2 min.

The centrifugation dewatering method has many advantages, including simplicity, speed of the process and its effectiveness. Centrifugation is useful for dewatering organic sludge [34,35] but also for dewatering mineral suspensions such as FFHCO2.

The increase in overload, not the centrifugation time (group a), was the cause of the slight increase in pH from 8.0 to 8.3 (Table 6). This change in chemical balance may be associated with a decrease in turbidity and conductivity, and thus changes in the concentrations of suspended particles and dissolved substances in the supernatant. The supernatant obtained after centrifugation at the lowest overload of rcf 1000 was characterized by a turbidity of approximately 150 NTU. In comparison, values of 300 NTU and higher were recorded for raw municipal wastewater [36]. Increasing the overload and centrifugation time resulted in a systematic reduction of turbidity to 89 NTU (9000 rcf, 1 min) and 68 NTU (9000 rcf, 2 min). The increase in overload during sample centrifugation resulted in a slight decrease in the conductivity of the supernatant and, therefore, in the amount of dissolved substances. However, this was not a function of centrifugation time. Overall, the average conductivity was approximately 360 µS/cm. This is not a high value, especially as the conductivity of municipal wastewater can range from 200 to 800 (µS/cm), depending mainly on the population served by the treatment plant [37].

The increase in overload and centrifugation time resulted in a quasi-linear decrease in iron concentration in the supernatant (Table 6). However, the amount of iron remaining in the supernatant was negligible. At baseline, the amount of iron in FFH was approximately 30 g/L, whereas in the supernatant it was only 3.0–1.5 mg/L. This can be compared to surface waters where the iron content generally does not exceed a few mg/L [38].

Solid–liquid separation of the FFHCO2 substrate by centrifugation may be recommended as a volume reduction method and as a method for recovery of ferrous compounds. The advisability of recovering iron compounds from FFHCO2 may depend on many factors, such as operating costs, the market value of the recovered iron and waste management laws and regulations. In any case, a cost–benefit analysis is required to assess whether iron recovery is cost effective and whether it is an appropriate strategy for the management of FFHCO2 [39].

### 3.2. Results of Stage II Tests

**Figure 5:**
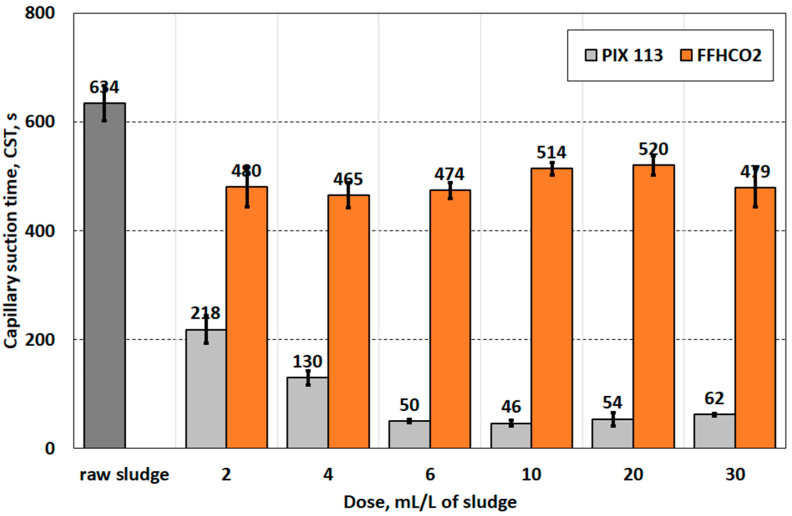
Comparison of capillary suction time of sludge conditioned with coagulant PIX 113 waste sorbent FFHCO2.

A capillary suction time test showed that the addition of FFHCO2 slightly improved the dewaterability of the sludge. It is important to note that increasing the dose from 2 to 30 mL/L does not reduce the CST value, which fluctuates between 460 and 520 s. Conversely, the use of iron coagulant PIX 113 allowed a gradual reduction of the capillary suction time from 630 (raw sludge) s to 50 s (6.0 mL PIX 113/L), (Figure 5).

**Figure 6:**
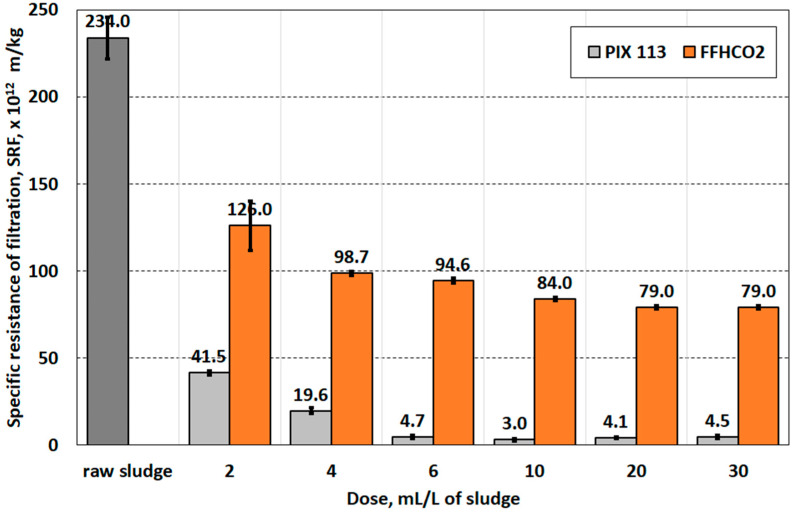
Specific filtration resistance of sludge conditioned with PIX 113 and FFHCO2.

Values of the specific resistance of filtration indicated that the use of FFHCO2 can improve the filtration capacity of sludge (Figure 6). However, the filtration resistance of sludge conditioned with FFHCO2 was significantly higher than 5.0 × 10^12^ m/kg, which is the threshold value for considering the sludge is susceptible to mechanical separation [32]. The PIX 113 dosage of 6.0 mL/L sludge already ensured effective dewatering of the conditioned sludge.

**Figure 7:**
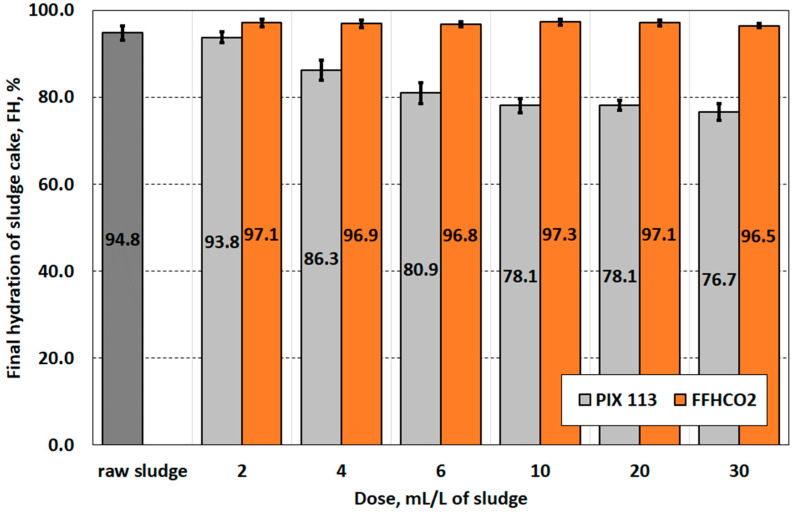
Final hydration of the filter cake after vacuum filtration of sludge conditioned with PIX 113 and FFHCO2.

The negative assessment of FFHCO2 as a sludge-conditioning agent prior to dewatering is confirmed by the results of filter cake hydration after sludge filtration (Figure 7). While the dosage of PIX 113 causes a gradual decrease in cake hydration, the dosage of FFHCO2 does not cause any significant changes. It is worth mentioning that filtration of raw sludge without conditioning allowed sludge dewatering to a level of 94.8%. Regardless of the dose of FFHCO2 used, hydration of the filter cake is around 97%. Xu et al. [40] and Zhang et al. [34] state that an effective conditioning agent must be able to reduce sludge hydration to below 80%. This condition was only met by using PIX 113 at a dose ≥10.0 g/L.

The addition of the coagulant PIX 113 (ferrous sulphate) caused a decrease in the pH of the supernatant after sludge filtration. On the contrary, FFHCO2 conditioning did not change the pH (Table 7). Different effects of PIX 113 and FFHCO2 conditioning on supernatant turbidity and conductance were also observed. The supernatant after filtration of PIX 113-conditioned sludge contained about three times fewer suspended particles and several times fewer dissolved particles compared to the use of FFHCO2 (Table 7). Testing the quality of supernatant after filtration is important for assessing the effectiveness of conditioning methods but also for ensuring compliance with environmental standards and minimizing negative impacts on the natural environment [41,42].

The parameters of the supernatant after the use of FFHCO2 indicate that the use of this type of sediment conditioner was not justified. In general, the effectiveness of FFHCO2 does not confirm its suitability as a coagulant. Nor does it state that FFH acts as a skeleton builder, similar to the addition of biochar, which effectively reduces sludge compressibility, increases permeability and releases bound water from the sludge structure [43].

### 3.3. Results of Stage III Tests

FFHCO2 was an alkaline substrate with a pH close to 9.0. The preparation of sample combinations C–F required mixing with acidic inoculum, which resulted in a decrease in pH to about 7.40 (Table 8). The addition of the energy substrate, i.e., ferrous sulphate, to the samples of combinations D and F resulted in a further decrease in pH. The method of sample preparation generally required the addition of significant amounts of concentrated sulfuric acid to acidify the samples to pH 2.0. Samples prepared by inoculation and dosing of FeSO_4_ and/or S^0^ (combinations C–F) showed a comparable sulfuric acid consumption of approximately 8.0 mL/L. The sample of combination B, i.e., waste FFHCO2 sorbent, required a dosage of almost 17 mL H_2_SO_4_/L.

**Figure 8:**
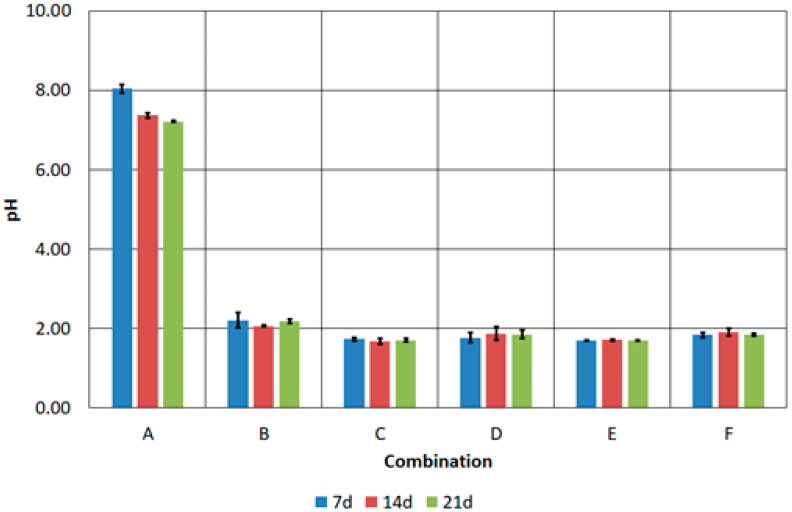
Changes in pH of prepared FFHCO2 samples.

**Figure 9:**
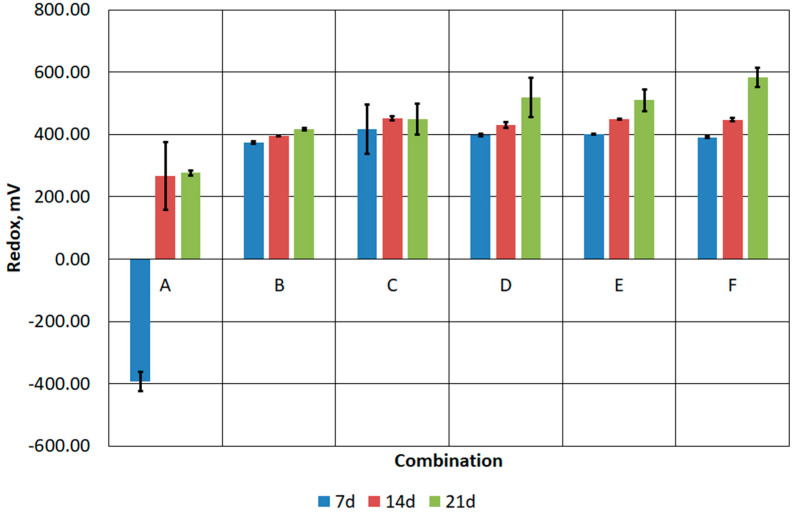
Changes in the redox potential of prepared FFHCO2 samples.

Pre-acidification allowed a low pH to be maintained in samples B-F throughout the duration of the experiment (Figure 8). A low pH, i.e., 2.0, is optimal for the development of bioleaching bacteria. Low pH affects the activity of microorganisms responsible for the degradation of organic contaminants and the solubility and mobility of metals in the environment [44,45]. Suitable conditions for the growth of *A. thiooxidans* and *A. ferrooxidans* were also confirmed by values of the oxidation–reduction potential [46,47] (Figure 9). The lowest redox value for the B–F combinations was about 400 mV and was recorded on the seventh day of the process. In each of the following 7 days of the process, the redox potential increased, even to values above 500 mV, which was observed in combinations D, E and F.

**Figure 10:**
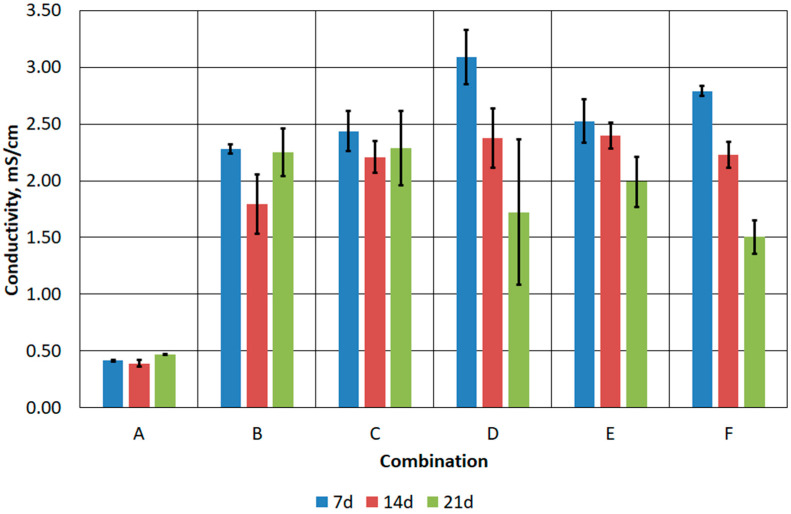
Changes in conductivity of prepared FFHCO2 samples.

During bioleaching, chemical and biochemical processes can affect the chemical composition of the solution, which can lead to changes in its conductivity. If an effective bioleaching process results in the release of metal ions from waste into the solution, an increase in conductivity can be expected due to the presence of these ions in the solution [48,49]. In the tested samples, higher conductivity was recorded in the samples subjected to acidification (Figure 10). In the case of combination A, the electrical conductivity did not exceed 0.5 mS/cm. In the case of combinations B–F, it ranged from 1.5 mS/cm to about 3.0 mS/cm. In addition, the general pattern for combinations B–F was a decrease in electrical conductivity with process time. However, the obtained conductance values were considered low, especially since the typical conductance value for sea water is 50 mS/cm [50].

**Figure 11:**
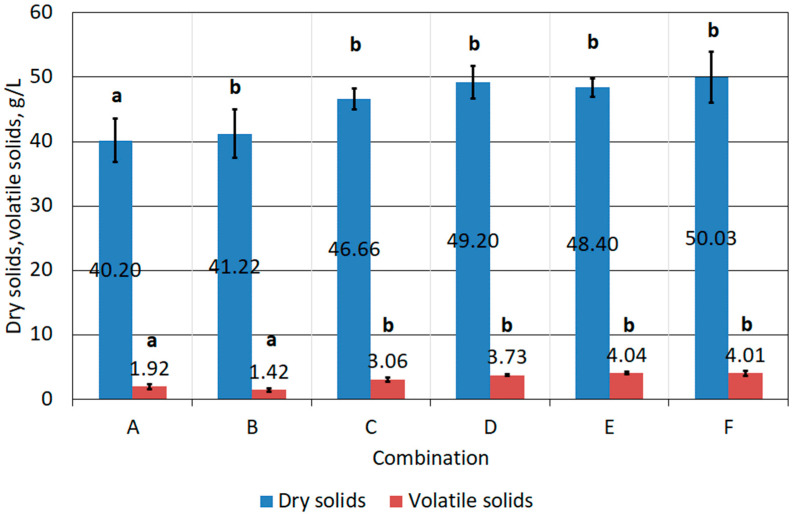
Mass changes of prepared FFHCO2 samples after 21-day incubation.

The 21-day bioleaching process did not result in a significant increase in dry solids and volatile solids in the tested samples (Figure 11). In combinations B–F, there was some increase in the dry solids of the samples, mainly due to dosing of the reagents. However, mathematical analysis showed that in combinations B–F (group b) changes in mass concentration were not statistically significant. An increase in volatile solids—organic matter—that could indicate an increase in biomass was observed in combinations C–F. It is concluded that only inoculated FFHCO2 samples, together with the addition of energy substrates (C–F), can potentially provide a suitable substrate for the growth of bioleaching bacteria. It should be noted that volatile solids (including biomass) represented a maximum of 8% of the dry solids of the samples (8% DS.). For comparison, the treatment of landfill leachate by bioleaching allowed a biomass yield of approximately 60% DS. (22 g/L) [51]. When water treatment sludge was used as a growth medium for bioleaching bacteria, the concentration of volatile solids—biomass—was approximately 46 g/L, which represented almost 80% of the dry mass of the sample [52].

In conclusion, there is no technological justification for the use of FFHCO2 as a substrate for the growth of bioleaching bacteria. The justification would be to meet the conditions for significant recovery of material, i.e., biomass, with low costs associated with sample preparation [53,54]. In the tests carried out, a 21-day process was required to achieve a low yield of organic matter, including dosing of inoculum with the nutrient solution, dosing of energy substrates and continuous aeration and heating.

After 21 days of incubation, the effective solid–liquid separation was also examined using capillary suction time and specific filtration resistance measurements. The recorded CST values of the samples of each combination were very similar. The CST ranged from 11 to 25 s. It was also found that the preparation method (combinations A–F) did not significantly affect the filtration capacity of the FFHCO2 samples (Table 9). The minimum value of r = 2.61 × 10^12^ m/kg was obtained for samples of combination A. The maximum value of approximately 4.5–5.0 × 10^12^ m/kg was obtained for samples of combinations C and F. In accordance with the standards for sewage sludge dewatering [31,32], obtained CSK and r values indicate that the tested sludge after bioleaching with FFHCO2 was an easily dewaterable substrate. Measurements of filtrate turbidity and conductance showed some differences depending on the method of sample preparation (Table 9). In the case of combinations E and F, where elemental sulfur was added to the samples, the filtrate was most contaminated with suspension and dissolved substances. The characteristics of the filtrate, regardless of the combination used, indicate that it must be subjected to further purification and/or recovery, similar to the filtrate from sludge dewatering [55].

The filtrate after the solid–liquid separation process was also assessed for the presence of iron and sulfur compounds as a potential mineral waste coagulant (iron sulphate), (Table 10). The lowest concentrations of sulfates and sulfites were found for combination A (67.5 mg/L SO_4_, 2.82 mg/L SO_3_). In combinations B–E, the concentration of sulfates was more than 100 times higher and sulfites about 50 times higher than in combination A. Iron content was also lowest in combination A (6.4 mg/L Fe), while in samples B–F it was 160–250 mg/L Fe.

The increased content of sulfates, sulfites and iron resulted from the dosage of energy substrates such as FeSO_4_ and S^0^ and from the activity of bioleaching microorganisms and ongoing oxidation and solubilization processes. An interesting aspect is the increase in sulfite concentration. The metabolism of bioleaching bacteria should lead to the oxidation of energy substrates to sulfates [56,57]. It can, therefore, be suggested that the samples were still a reaction–culture environment. On the other hand, it is equally probable that _cation_ concentrations had an inhibitory effect on metabolism and biomass growth [27,56]. The demand for innovative products, sustainable technologies and a closed-loop economy are increasingly treated as an obligation rather than a necessity. The main drivers of profitability and efficiency are slowly paving the way for products to have a negative impact on the environment. This approach is observed especially in polymeric materials, where attention is focused on tools to measure and reduce the negative environmental impact of materials throughout their life cycle, the use of renewable sources for their synthesis, the design of biodegradable and/or recyclable materials and the use of biotechnology strategies for enzymatic recycling, which fit into the closed-loop bioeconomy. Conscious consumption behavior is becoming an important aspect as well as reducing the impact of the supply chain and reducing the energy and material intensity of products, ultimately promoting a holistic life-cycle approach to reduce overall waste and pollution [58].

## 4. Conclusions

The use of FFHCO2 as a sludge conditioner prior to dewatering did not improve the technological properties of the wastewater sludge. The tests carried out indicate that FFHCO2 binds the water contained in the structure of the sludge and limits their filterability. There is no reason to classify FFHCO2 as an iron waste coagulant or even as a structure-forming agent to support solid–liquid separation.

Experiments on the treatment of spent iron sorbent by bioleaching are critically evaluated. The maximum achievable organic matter content in the samples tested was about 8% of dry solids. It is concluded that FFHCO2 is not a substrate for the effective growth of the biomass of leaching microorganisms. The disadvantage of the FFHCO2 bioleaching process is also the need for FFHCO2 inoculation, the addition of culture medium, the addition of energy substrates and a 21-day culture requiring aeration and heating.

Solid–liquid separation after the FFHCO2 bioleaching process was not a technological problem. The obtained parameters of the solid phase and filtrate did not indicate their possible application potential. The production of products with coagulating or sorption properties by bioleaching FFHCO2 was considered a method with no prospect of practical application.

The most simple and fast method for pre-processing waste FFHCO2 sorbent was centrifugation. The high degree of solid–liquid separation enabled the recovery of the solid phase containing iron compounds, which is the basis for further processing or disposal processes.

It is concluded that the basic strategy for dealing with waste FFHCO2 sorbent is to minimize the amount of waste by volume reduction—dewatering. Increasing interests toward the implementation of circular economy rules by, e.g., utilizing wasted materials to produce new materials is steadily developing. In order to be in agreement with the circular economy approach, it is proposed to recover material such as iron compounds from waste and to utilize it for other processes such as the production of new materials rich in iron compounds for, e.g., chemical, agricultural or construction industries. However, such an approach needs further investigation.

## Figures and Tables

**Table 1:** Basic characteristics of FFHCO2.

Dry solids, DS, g/L	45.30 ± 0.30
Volatile solids, VS, g/L	4.95 ± 0.22
pH	8.80 ± 0.1
Alkalinity, mg CaCO_3_/L	28,000.0 ± 707.0
Magnetic field lines marked by FFHCO2 particles. (Stir bar, diameter × length: 0.7 × 40 mm.)	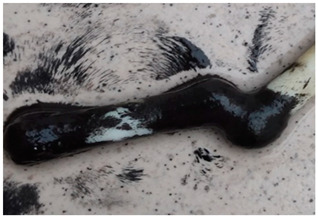

**Table 2:** Physicochemical properties of tested stabilized sludge.

Dry solids, DS g/L	22.6 ± 0.6
Volatile solids, VS g/L	17.3 ± 0.7
pH	6.40 ± 0.21
Capillary suction time (CST), s	634.0 ± 32.0
Specific resistance of filtration, (SRT), E12 m/kg	234 ± 1.2
Final hydration of sludge cake (FH), %	94.8 ± 1.6

**Table 3:** Basic characteristics of the inoculum (biomass + K9).

Dry Solids, g/L	Volatile Solids, g/L	Volatile Solids Content, % DS	pH
10.92 ± 0.34	8.03 ± 0.94	≈73.5	1.81 ± 0.12

**Table 4:** Research combinations of FFH processing by bioleaching.

Combination	A	B	C	D	E	F
FFH, % of sample volume	100	100	50	50	50	50
Inoculum, % of sample volume	0	0	50	50	50	50
pH correction to		2.0	2.0	2.0	2.0	2.0
Addition of FeSO_4_·7H_2_O, g/L				44.0		44.0
Addition of S^0^, g/L					10.0	10.0

**Table 5:** Centrifugation efficiency as the percentage of solid phase separated from the tested suspension FFHCO2.

Centrifugation Efficiency, %							Statistical Group
Centrifugation Time, min.	1 min.	98.3	98.3	98.5	98.7	98.8	a
	2 min.	98.5	98.6	98.8	98.9	99.0	a

**Table 6:** Supernatant parameters after centrifugation.

Parameter	Centrifugation Time	1000	3000	5000	7000	9000	Statistical Group
pH	1 min.	8.0 ± 0.1	8.2 ± 0.1	8.2 ± 0.1	8.3 ± 0.2	8.3 ± 0.12	a
	2 min.	8.0 ± 0.2	8.1 ± 0.1	8.2 ± 0.1	8.2 ± 0.1	8.3 ± 0.2	a
Turbidity, NTU	1 min.	157 ± 10	153 ± 15	138 ± 14	100 ± 11	89 ± 8	a
	2 min.	137 ± 18	121 ± 19	96 ± 12	78 ± 9	68 ± 12	b
Conductance, uS/cm	1 min.	374 ± 32	380 ± 17	358 ± 23	350 ± 26	353 ± 13	a
	2 min.	368 ± 26	364 ± 29	344 ± 21	348 ± 11	346 ± 17	a
Total iron content, mg Fe/L	1 min.	3.0 ± 0.34	2.8 ± 0.14	2.3 ± 0.25	1.8 ± 0.18	1.5 ± 0.11	a
	2 min.	2.6 ± 0.24	1.9 ± 0.15	1.8 ± 0.11	1.5 ± 0.26	1.4 ± 0.09	b

**Table 7:** Supernatant parameters after filtration of sludge conditioned with PIX 113 and FFHCO2.

Parameter	Conditioner	Dose of Conditioner, mL/L of Sludge						Statistical Group
		2	4	6	10	20	30	
pH	PIX 113	5.39 ± 0.06	4.87 ± 0.11	4.47 ± 0.11	3.03 ± 0.14	2.44 ± 0.06	2.27 ± 0.09	a
	FFHCO2	5.99 ± 0.06	6.0 ± 0.09	5.99 ± 0.11	6.01 ± 0.08	6.09 ± 0.19	6.06 ± 0.10	b
Turbidity NTU	PIX 113	216 ± 11	199 ± 10	187 ± 13	151 ± 15	144 ± 9	137 ± 3	a
	FFHCO2	487 ± 36	506 ± 29	531 ± 26	536 ± 24	473 ± 24	529 ± 27	b
Conductance uS/cm	PIX 113	169 ± 11	150 ± 7	140 ± 10	104 ± 8	77 ± 11	57 ± 9	a
	FFHCO2	1191 ± 28	1569 ± 33	2239 ± 43	2275 ± 28	2038 ± 58	2005 ± 68	b

**Table 8:** Parameters of FFHCO2 samples prepared before the bioleaching process.

Combination	A	B	C	D	E	F
Initial pH	8.80	8.80	8.80	8.80	8.80	8.80
pH after inoculation	-	-	7.40 ± 0.06	7.39 ± 0.09	7.37 ± 0.05	7.38 ± 0.10
pH after adding FeSO_4_	-	-	-	6.96 ± 0.10	-	7.06 ± 0.09
Amount of sulfuric acid, pH correction, mL/L	-	16.8 ± 0.3	8.00 ± 0.2	7.8 ± 0.1	8.0 ± 0.2	7.8 ± 0.3
Final pH	8.80	2.0	2.0	2.0	2.0	2.0

**Table 9:** Solid–liquid separation parameters of FFHCO2 after the bioleaching process.

Combination	A	B	C	D	E	F
CSK, s	11 ± 5	16 ± 2	20 ± 5	17 ± 5	15 ± 4	25 ± 3
r, 10E12 m/kg	2.61 ± 0.57	3.71 ± 0.81	4.49 ± 0.98	3.85 ± 0.73	3.35 ± 0,73	4.92 ± 0.72
Turbidity (filtrate), NTU	12.9 ± 1.3	6.92 ± 0.7	11.3 ± 3.2	10.3 ± 2.0	31.5 ± 7.6	26.5 ± 3.1
Conductance (filtrate), mS/cm	0.316 ± 0.09	0.879 ± 0.15	0.710 ± 0.42	0.483 ± 0.11	1.709 ± 0.26	1.642 ± 0.33

**Table 10:** Characteristics of the filtrate after the FFHCO2 separation process after bioleaching.

Combination	LCK 153	LCK 321	LCK 654
	mg/L SO_4_	mg/L Fe	mg/L SO_3_
**A**	67.5 ± 5	6.4 ± 0.4	2.8 ± 0.3
**B**	669 ± 49	160.3 ± 1.2	123.7 ± 1.9
**C**	7280 ± 35	174.6 ± 8.6	80.3 ± 2.7
**D**	8970 ± 61	214.5 ± 5.2	126.4 ± 3.4
**E**	8030 ± 85	194.0 ± 7.0	108.6 ± 1.9
**F**	10,510 ± 66	250.1 ± 14.6	135.0 ± 5.9

## Data Availability

The original contributions presented in the study are included in the article, further inquiries can be directed to the corresponding authors.
